# Association between prebiotic, probiotic consumption and hyperuricemia in U.S. adults: a cross-sectional study from NHANES 2011–2018

**DOI:** 10.3389/fnut.2025.1492708

**Published:** 2025-03-14

**Authors:** Yixuan Wang, Shiwei Li, Xin Li, Meng Wang, Bo Huang, Kailei Feng, Jingqiu Cui

**Affiliations:** Department of Endocrinology and Metabolism, Tianjin Medical University General Hospital, Tianjin, China

**Keywords:** prebiotics, probiotics, hyperuricemia, intestinal flora, NHANES

## Abstract

**Aim:**

This study aims to provide evidence for an association between the consumption of prebiotics and probiotics and hyperuricemia in U.S. adults.

**Methods:**

A total of 7,176 adults who participated in the National Health and Nutrition Examination Survey (NHANES) during 2011–2018 were included in the study. First, the baseline characteristics of the data were described for the weighted data, using the presence or absence of hyperuricemia as the classification criterion. Second, binary logistic regression analyses were performed to establish crude models and regression models adjusted for relevant covariates, and odds ratios (OR) and 95% confidence intervals (95% CI) were calculated to explore the relationship between prebiotics, probiotic intake, and hyperuricemia. Subsequently, receiver operating characteristic (ROC) curves were plotted to assess probiotic consumption’s role in the hyperuricemia prediction model. Finally, subgroup analyses were performed.

**Results:**

Participants who consumed probiotics had a lower prevalence of hyperuricemia than those who did not (3.48% vs. 6.25%, *p* = 0.082). In logistic regression analyses, prebiotics’ effect on hyperuricemia was insignificant (*p* > 0.05), regardless of whether covariates were considered. In contrast, the crude model for probiotics and the adjusted model 1, which was constructed by adjusting for age, sex, and ethnicity, showed ORs less than 1 (crude model: OR = 0.54, 95% CI [0.34, 0.83], *p* = 0.008; adjusted model 1: OR = 0.54, 95% CI [0.34, 0.83], *p* = 0.008). The predictive model, including age, sex, race, body mass index (BMI), hypertension, chronic kidney disease, and probiotics, had 76.7% sensitivity and 68.0% specificity with an area under the ROC curve of 0.7886 for detecting hyperuricemia in US adults.

**Conclusion:**

These results suggest that probiotic consumption may reduce the incidence of hyperuricemia in the US adult population, but prebiotics have not shown the same effect.

## 1 Introduction

Uric acid (UA) is synthesized primarily in the liver, intestine, and vascular endothelium. Hyperuricemia (HUA) occurs when UA production exceeds its excretion, a condition regarded as a metabolic disorder. It is generally defined as a serum urate level exceeding 7.0 mg/dL in men or 6.0 mg/dL in women (1). The well-documented consequence of hyperuricemia is an increased risk of gout and the formation of kidney stones. Approximately 20% of the United States population is affected by hyperuricemia (2). Statistically, the prevalence of hyperuricemia is significantly higher in men than in women (3, 4). The etiology of hyperuricemia is multifactorial, encompassing a range of acquired factors. These primarily include increased purine consumption due to dietary intake of meat, alcohol, high-fructose corn syrup, and certain medications such as cyclosporine, low-dose aspirin, and diuretics. Additionally, rare genetic factors can contribute to the condition, including hypoxanthine-guanine phosphoribosyl transferase (HPRT) deficiency and phosphoribosyl pyrophosphate (PRPP) synthase overactivity. Furthermore, myeloproliferative disorders and environmental exposures, such as lead poisoning, temperature fluctuations, and physiological stress, may also lead to elevated uric acid levels (5).

In healthy individuals, approximately 25% of uric acid is excreted through the gastrointestinal tract, in contrast to its renal excretion. Exposure of the gut microbiota to uric acid (UA) may alter the composition of the microbiota (6). Gut microbiota and their metabolites can reduce serum uric acid (SUA) levels by promoting purine and UA catabolism, modulating intestinal UA transporter proteins, enhancing UA excretion, and regulating intestinal barrier permeability to mitigate chronic inflammation (7). Probiotics is a topic of much debate in contemporary scientific discourse. Probiotics are health-promoting products, typically lactic acid-producing bacteria introduced through fermented foods, such as *Lactobacillus* and *Bifidobacterium* (8). These bacteria have been shown to benefit human health by preventing or treating diseases and restoring the balance of intestinal flora through mechanisms such as antimicrobial action, enhancement of the integrity of mucosal barriers, and immunomodulation (9, 10). Clinical findings confirm the positive effects of probiotics on gastrointestinal disorders (e.g., inflammatory bowel disease, diarrhea) and immune disorders (e.g., HIV), and studies have also demonstrated the efficacy of probiotics for the treatment of conditions such as obesity, insulin resistance syndrome, type 2 diabetes mellitus, and non-alcoholic fatty liver disease (8, 11). Prebiotics are defined as non-viable food components that confer a health benefit to the host associated with the microbiota modulation. This definition is regarded as the most authoritative to date by the Food and Agriculture Organisation of the United Nations (FAO) (12). It is hypothesized that prebiotics have the capacity to modify the gut microbiota, with distinct prebiotics promoting the proliferation of various gut-colonizing bacteria. However, these alterations are frequently constrained at the level of individual strains and species, rendering them challenging to predict *a priori* (13). The World Health Organization (WHO), the Food and Agriculture Organization (FAO), the European Food Safety Authority (EFSA), the International Scientific Association for Probiotics and Prebiotics (ISAPP), and other organizations have provided comprehensive classifications of edible prebiotics and probiotics for reference (13, 14).

Despite numerous studies investigating how probiotics and prebiotics modulate the gut microbiota in patients with HUA, extensive cross-sectional studies still fail to assess the risk of HUA prevalence in populations consuming these supplements. Therefore, this study aims to analyze the relationship between probiotic and prebiotic supplements and the prevalence of HUA in U.S. adults. This analysis utilizes data from the National Health and Nutrition Examination Survey (NHANES) from 2011 to 2018 and evaluates whether potential confounders affect this association.

## 2 Materials and methods

### 2.1 Study design and population

The National Health and Nutrition Examination Survey (NHANES) is a nationally representative cross-sectional study conducted by the National Center for Health Statistics (NCHS) to assess adults’ and children’s health and nutritional status in the United States. Data collection and study approaches utilize a sophisticated multistage probability sampling design. The information gathered is used to evaluate nutritional status and its relationship to health promotion and disease prevention, providing valuable insights into the prevalence of major diseases and associated risk factors. The NCHS Ethical Review Committee authorized all investigations, and all participants provided informed consent.

**Figure 1:**
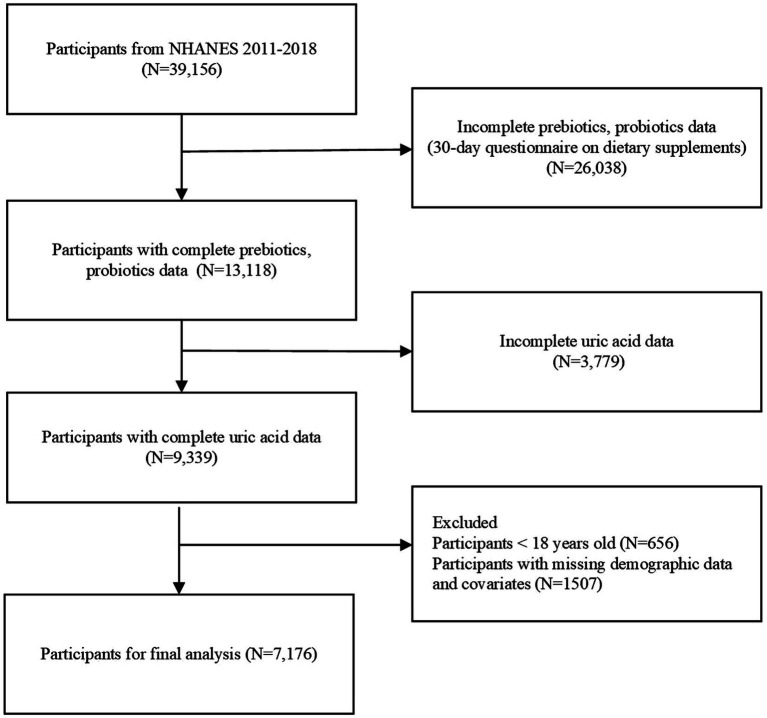
Flow chart of procedures for selection and inclusion of participants. NHANES, National Health and Nutrition Examination Survey.

The purpose of this study was to conduct a cross-sectional analysis of hyperuricemia (HUA) patients using data from the National Health and Nutrition Examination Survey (NHANES) from 2011 to 2018. The NHANES database is regularly updated, and a nationally representative sample of approximately 5,000 individuals is examined annually. The following exclusion criteria were applied: age under 18 years, absence of data on uric acid levels, absence of data on non-dietary prebiotics/probiotics intake, absence of demographic data (including age, gender, race/ethnicity, education level, poverty income ratio), absence of data on alcohol status, creatinine, body mass index (BMI), hypertension (HBP), and diabetes mellitus (DM). A total of 7,176 participants were ultimately included in the analysis. Figure 1 presents a flowchart outlining the participant selection and inclusion process. These data were then used to assess the relationship between probiotics or prebiotics and the risk of hyperuricemia.

### 2.2 The diagnosis of hyperuricemia

There is no consensus on the definition of hyperuricemia, and different authorities have chosen different thresholds. In this paper, concerning previous studies, a threshold of 420 μmol/L has been chosen to define hyperuricemia. This value is considered the possible limit for clinical gout and corresponds to the serum uric acid (SUA) target for uric acid-lowering therapy in patients with gout (15, 16).

### 2.3 Assessment of probiotic and prebiotic

We collected data on probiotics and prebiotics supplements from all NHANES annual cycles from 2011 to 2018. Scholar O′Connor’s literature summarizes a list of names for prebiotics and probiotics, aiding in identifying specific species (details in Supplementary Table S1) (17). Based on the results of a 30-day questionnaire on dietary supplements, we collected information on the use of dietary supplements during the 30 days prior to the interview to assess the intake of dietary supplements containing prebiotics and probiotics.

### 2.4 Definition of co-variables

Demographic characteristics for inclusion in this study included age, gender, race (Non-Hispanic White, Non-Hispanic Black, Hispanic, other Hispanic, other Race – Including Multi-Racial), body mass index (BMI), poverty-to-income ratio (PIR), and education level. Participants were categorized into underweight/normal weight (BMI < 25 kg/m^2^), overweight (BMI = 25–30 kg/m^2^), and obese (BMI > 30.0 kg/m^2^) according to WHO standards. The PIR was categorized into three tiers: low-income households (PIR < 1.3), moderate household income (PIR of 1.3–3.5), and high household income (PIR > 3.5). Education level was divided into three categories: below high school level, high school level, and above high school level (18). Additionally, alcohol consumption status, hypertension (HBP), and diabetes mellitus (DM) were included as covariates in the form of yes or no. Chronic kidney disease (CKD) was defined as an estimated glomerular filtration rate (eGFR) of less than 60 mL/min/1.73 m^2^, with eGFR values directly retrievable from the database. The modified MDRD equation was used to calculate eGFR with the following formula:


$$\mathrm{eGFR}=186\times {\left(Scr\right)}^{-1.154}\times \mathrm{ag}e^{-0.203}\times 0.742^{\mathrm{if}\;\mathrm{female}}$$

(Details can be found in the NHANES database definitions for each).

### 2.5 Statistical analysis

To ensure the reliability and validity of the study results, NHANES conducted a stratified sample with each cycle lasting 2 years, resulting in 4 cycles over an 8-year period. For the dietary recall interview, dietary data were weighted using the corresponding dietary weight for each cycle (WTDR2D). Given the proportional representation of each cycle in the survey design, the weight for each cycle was calculated as 2/8*WTDR2D. We analyzed the data using appropriate sampling weights (1/4*WTDR2D) to account for the complex survey design used in the NHANES survey. In this survey, variables were categorized and expressed as frequencies and percentages. We also compared baseline characteristics between participants with positive and negative outcomes using *χ*^2^ test analyses.

We divided participants into those who used prebiotics and probiotics and those who did not use either. We fitted binary logistic regression models, adjusting for covariates, to assess the relationship between prebiotic and probiotic consumption and the risk of HUA, presented as an odds ratio (OR) with a 95% confidence interval (95% CI). We then fitted several models to explore the possible association between prebiotic and probiotic consumption and HUA: an unadjusted model, adjusted model 1 (adjusted for age, gender, and ethnicity), and adjusted model 2 (adjusted model 1 plus BMI, education, CKD, HBP, and DM). We considered prebiotics and probiotics as interacting factors in further modeled analyses of the results.

Receiver operating characteristic (ROC) curves are commonly used to assess the performance of binary classification models. In this study, we used ROC to assess the performance of different prediction models in predicting hyperuricemia. The maximal model included the following variables: age, gender, race, education level, PIR, alcohol status, BMI, CKD, HBP, DM, and probiotics. The candidate model included age, gender, race, BMI, CKD, HBP, and probiotics. To determine if there was a significant difference between the two predictive models, we used a *z*-test to compare the area under the ROC curve (AUC) for each model. In addition, we added a model without probiotics to further clarify the predictive performance of probiotics for comparison with the candidate models.

Subgroup analyses were performed to examine the potential impact on the association between probiotic consumption and hyperuricemia of gender, age, ethnicity, education level, BMI, CKD, HBP, and DM.

Statistical analysis was performed using R version 4.4.0 and the nhanesR package, and *p* < 0.05 was considered statistically significant.

## 3 Results

### 3.1 Descriptive characteristics

The sample for this study was selected from data collected by NHANES during 2011–2018, and 7,176 participant records were ultimately included in the analysis. Table 1 shows the population stratified by the presence or absence of hyperuricemia and demographic characteristics, of which 859 (16.0%) were prevalent. All participants over 18 were selected for the study, and the mean age of the recipients was approximately 50.95 years. As shown in Table 1, patients with hyperuricemia were generally older (53.80 ± 17.33 vs. 50.60 ± 17.00), had a higher BMI (32.28 ± 7.09 vs. 28.61 ± 6.63), were more common in the male population (74.96% in the male population vs. 25. 04% in the female population), a slightly higher proportion of Non-Hispanic White participants (76.17%), and a slightly lower level of education, as evidenced by a lower percentage of the population with more than high school education (66.99% vs. 70.92%) and a higher percentage of high school education (23.20% vs. 18.69%). Regarding disease status, eGFR was lower in patients with hyperuricemia (73.76 ± 23.09 vs. 86.92 ± 22.13). There was a more frequent prevalence of hyperuricemia in patients with chronic kidney disease, hypertension, and diabetes mellitus (26.61% vs. 9.35% for chronic kidney disease, 63.29% vs. 40.01% for hypertension, and 23.82% vs. 15.34% for diabetes mellitus). All these results were statistically significant (*p* < 0.05). In addition, people with hyperuricemia were more likely to smoke (90.66% vs. 89.05%) and had relatively less family poverty (3.19 ± 1.59 vs. 3.24 ± 1.61), but unfortunately, these results were not statistically significant.

**Table 1:** Descriptive characteristics of the study population stratified by hyperuricemia.

Characteristic	Overall, *N* = 7,176 (100%)^1^	Hyperuricemia		*P*-value^2^
		No, *N* = 6,317 (84%)^1^	Yes, *N* = 859 (16%)^1^	
Age (years)	50.95 ± 17.07	50.60 ± 17.00	53.80 ± 17.33	<0.001
BMI (kg/m^2^)	29.02 ± 6.78	28.61 ± 6.63	32.28 ± 7.09	<0.001
eGFR (ml/min/1.73 m^2^)	85.46 ± 22.61	86.92 ± 22.13	73.76 ± 23.09	<0.001
PIR	3.23 ± 1.61	3.24 ± 1.61	3.19 ± 1.59	0.4
Gender, *n* (%)				<0.001
Female	3,955.00 (55.00%)	3,717.00 (58.73%)	238.00 (25.04%)	
Male	3,221.00 (45.00%)	2,600.00 (41.27%)	621.00 (74.96%)	
Race/ethnicity, *n* (%)				0.001
Mexican American	781.00 (5.59%)	721.00 (5.86%)	60.00 (3.41%)	
Non-Hispanic Black	1,312.00 (7.87%)	1,105.00 (7.64%)	207.00 (9.70%)	
Non-Hispanic White	3,335.00 (74.45%)	2,927.00 (74.24%)	408.00 (76.17%)	
Other Hispanic	665.00 (4.34%)	600.00 (4.44%)	65.00 (3.55%)	
Other Race – Including Multi-Racial	1,083.00 (7.74%)	964.00 (7.82%)	119.00 (7.17%)	
Education levels, *n* (%)				0.013
More than high school	4,503.00 (70.49%)	3,991.00 (70.92%)	512.00 (66.99%)	
High school graduate/GED or equivalent	1,476.00 (19.18%)	1,274.00 (18.69%)	202.00 (23.20%)	
<High school	1,197.00 (10.33%)	1,052.00 (10.39%)	145.00 (9.80%)	
Alcohol consumption, *n* (%)				0.3
No	1,056.00 (10.77%)	958.00 (10.95%)	98.00 (9.34%)	
Yes	6,120.00 (89.23%)	5,359.00 (89.05%)	761.00 (90.66%)	
HBP, *n* (%)				<0.001
No	3,805.00 (57.42%)	3,526.00 (59.99%)	279.00 (36.71%)	
Yes	3,371.00 (42.58%)	2,791.00 (40.01%)	580.00 (63.29%)	
DM, *n* (%)				<0.001
No	5,697.00 (83.72%)	5,104.00 (84.66%)	593.00 (76.18%)	
Yes	1,479.00 (16.28%)	1,213.00 (15.34%)	266.00 (23.82%)	
CKD, *n* (%)				<0.001
No	6,307.00 (88.74%)	5,709.00 (90.65%)	598.00 (73.39%)	
Yes	869.00 (11.26%)	608.00 (9.35%)	261.00 (26.61%)	
Prebiotics, *n* (%)				0.4
No	6,805.00 (93.80%)	5,985.00 (93.68%)	820.00 (94.76%)	
Yes	371.00 (6.20%)	332.00 (6.32%)	39.00 (5.24%)	
Probiotics, *n* (%)				0.082
No	6,876.00 (94.06%)	6,038.00 (93.75%)	838.00 (96.52%)	
Yes	300.00 (5.94%)	279.00 (6.25%)	21.00 (3.48%)	

^1^Median (IQR) for continuous; n (%) for categorical.

^2Wilcoxon rank-sum test for complex survey samples; chi-squared test with Rao and Scott’s second-order correction.^

*P-value by chi-square test for classified variables.*

BMI, body mass index; CKD, chronic kidney disease; DM, diabetes mellitus; HBP, high blood pressure; PIR, poverty income ratio; eGFR, estimated glomerular filtration rate.

### 3.2 Binary logistic regression analysis

A crude model and two other logistic regression models adjusted for covariates were developed to investigate whether prebiotic and probiotic consumption had a preventive effect on hyperuricemia, as shown in Table 2. The ORs of the prebiotic-related models were all less than 1 but were not significant after statistical testing (*p* > 0.05). The ORs calculated for both the crude model and adjusted model 1 for probiotics and hyperuricemia were also less than 1 and tested to be statistically significant (crude model: OR = 0.54, 95% CI [0.34, 0.83], *p* = 0.008; adjusted model 1: OR = 0.58, 95% CI [0.36, 0.89], *p* = 0.019). However, when we increased the number of covariates to create adjusted model 2, it had an OR of 0.65, 95% CI (0.41, 1.00) with a tested *p*-value above 0.05 (0.062).

**Table 2:** Binary logistic regression models for the association of consuming probiotics and prebiotics with hyperuricemia (HUA).

Characteristic	Unadjusted model	Adjusted model 1	Adjusted model 2
	OR^1^ (95% CI^1^) associated with HUA		
Prebiotics			
No	1 (Ref)	1 (Ref)	1 (Ref)
Yes	0.86 (0.60, 1.19); 0.374	0.79 (0.55, 1.09); 0.167	0.85 (0.59, 1.18); 0.384
Probiotics			
No	1 (Ref)	1 (Ref)	1 (Ref)
Yes	0.54 (0.34, 0.83); 0.008	0.58 (0.36, 0.89); 0.019	0.65 (0.41, 1.00); 0.062

^1OR, Odds Ratio; CI, Confidence Interval.^

Unadjusted model: non-adjusted model. Adjust model 1: adjust for age, gender and race. Adjust model 2: adjust for age, gender, race, body mass index, education levels, chronic kidney disease, diabetes mellitus, hypertension, prebiotics, and probiotics.

To further investigate whether the consumption of probiotics and prebiotics interacted with each other on hyperuricemia, we defined prebiotics/prebiotics as the consumption of prebiotics, probiotics, or both, and constructed three logistic regression models, as shown in Table 3. Compared to participants who consumed neither prebiotics nor probiotics, participants who consumed prebiotics and probiotics alone or both had ORs less than 1 after model fitting, demonstrating a protective effect, but this was tested to be statistically significant only in the models with probiotics alone (crude model for probiotics: OR = 0.45, 95%CI [0.23, 0.79], *p* = 0.011; Adjust model 1: OR = 0.52, 95%CI [0.27, 0.92], *p* = 0.037; Adjust model 2: OR = 0.58, 95%CI [0.30, 1.03], *p* = 0.079). In conclusion, these results suggest that the intake of prebiotics and probiotics has a protective effect against hyperuricemia, but the impact of probiotics is more valuable.

**Table 3:** Binary logistic regression models for the association of consuming prebiotics/probiotics with hyperuricemia (HUA).

Characteristic	Unadjusted model	Adjusted model 1	Adjusted model 2
	OR^1^ (95% CI^1^) associated with HUA		
Prebiotics/probiotics			
Neither of them	1 (Ref)	1 (Ref)	1 (Ref)
Just prebiotics	0.91 (0.60, 1.32); 0.636	0.83 (0.55, 1.21); 0.340	0.87 (0.58, 1.28); 0.500
Just probiotics	0.45 (0.23, 0.79); 0.011	0.52 (0.27, 0.92); 0.037	0.58 (0.30, 1.03); 0.079
Both of them	0.70 (0.34, 1.27); 0.279	0.66 (0.33, 1.22); 0.218	0.75 (0.37, 1.39); 0.388

^1OR, Odds Ratio; CI, Confidence Interval.^

Unadjusted model: non-adjusted model. Adjust model 1: adjust for age, gender and race. Adjust model 2: adjust for age, gender, race, body mass index, education levels, chronic kidney disease, diabetes mellitus, hypertension, prebiotics, and probiotics.

Probiotics/prebiotics refer to either probiotics, prebiotics, or both.

### 3.3 Receiver operating characteristic curve

**Figure 2:**
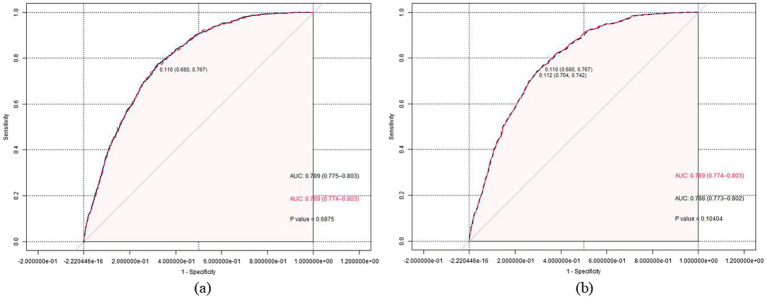
**(a)** The receiver operating characteristic (ROC) curves for the maximum (black dashed line) and the candidate (red solid line) prediction model are presented. The *Z*-test of the area under the ROC curve did not show a significant difference in the predictive performance of the two models for periodontitis (*Z* = 0.402, *p* = 0.688). **(b)** The ROC curves for the candidate prediction model (red solid line) and the prediction model without prebiotic (black dashed line) are presented. The *Z*-test of the area under the ROC curve did not show a significant difference in the predictive performance of the two models for periodontitis (*Z* = 1.626, *p* = 0.104).

Based on the non-significant difference in the prevalence of hyperuricemia between participants who consumed prebiotics and those who did not, the results provided tentative support for a notable association with hyperuricemia, and we decided not to include prebiotics in the prediction model. The prediction model with the highest AUC value (AUC = 0.7889, 95%CI [0.775, 0.803], sensitivity = 0.774, specificity = 0.106) included the following variables: age, sex, race, BMI, educational level, PIR, alcohol status, HBP, DM, CKD, and probiotics. However, this maximum model includes too many variables, compromising its clinical applicability and not considering the AIC criteria (19). We sought to identify a more parsimonious prediction model to address this issue while maintaining similar predictive performance. When considering models with six or fewer variables, we observed a significant reduction in predictive performance compared to the maximum model of all models. Finally, when we adjusted the predictive model to include seven variables (including age, sex, race, BMI, HBP, CKD, and probiotics), the model showed similar predictive performance to the maximum model (AUC = 0.7886, 95% CI [0.774, 0. 803], sensitivity = 0.767, specificity = 0.680), and the ROC curves of the candidate and maximal models were similar, the *z*-test of AUC showed no significant difference in the predictive performance of the two models for HUA (*z* = 0.402, *p* = 0.680), as shown in Figure 2a. In Figure 2b, we removed probiotics from the candidate model, and the resulting predictive model showed a significant reduction in AUC compared to the candidate model, but this difference was not tested to be statistically significant (0.7877 versus 0.7886, *Z* = 1.626, *p* = 0.104). This suggests that probiotics might positively improve the diagnostic performance of the predictive model for hyperuricemia, but this effect was not noticeable.

### 3.4 Subgroup analysis

**Figure 3:**
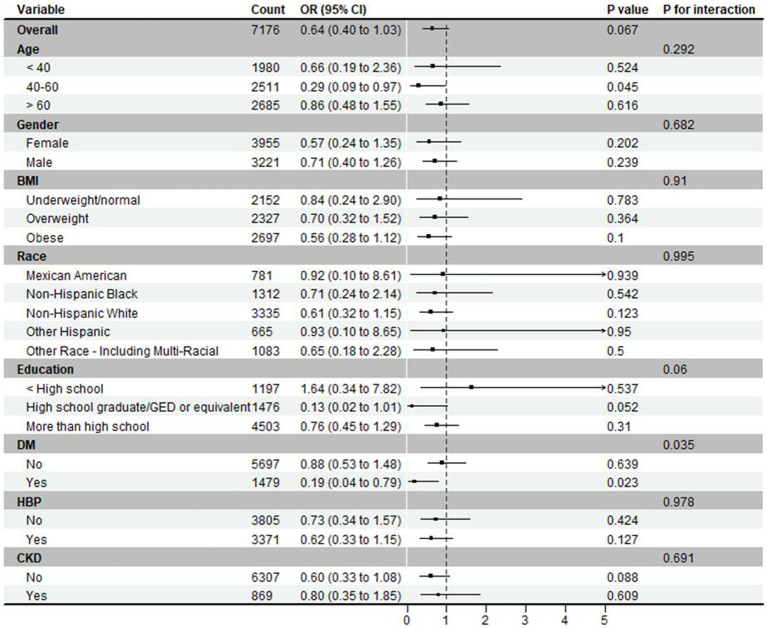
Subgroup analysis and forest plot on hyperuricemia.

Subgroup analysis was performed on the ORs for developing hyperuricemia in those receiving probiotics compared to those not (Figure 3). All analyses were adjusted for age, sex, race, body mass index, education level, hypertension, diabetes mellitus, and chronic kidney disease but not for the specific stratification variables of interest. Except for diabetes, all subgroups had interaction *p*-values greater than 0.05, indicating no statistically significant effect modification. Thus, the association between probiotics and hyperuricemia remained consistent across the different subgroups defined by these variables.

## 4 Discussion

This study is a comprehensive, large-scale, cross-sectional investigation of the association between prebiotic and probiotic consumption and the development of hyperuricemia in US adults. To ensure independence of the association between exposure and outcome, several covariates were included in the analyses, including age, sex, race, BMI, PIR, education level, alcohol consumption status, hypertension, diabetes, and chronic kidney disease. The results of the logistic regression models showed that both the prebiotic- and probiotic-related models calculated ORs of less than 1 regardless of the inclusion of covariates, but only the probiotic-related models were statistically different after testing (crude model: OR = 0.54, 95% CI [0.34, 0.83], *p* = 0.008; adjusted model 1: OR = 0.58, 95% CI [0.36, 0.89], *p* = 0.019). This finding shows that probiotic intake may reduce the incidence of hyperuricemia. After we classified the intake of prebiotics and probiotics in detail, further logistic regression analysis suggested that the prebiotic-related model still showed no significant difference but that the protective effect of probiotics alone on hyperuricemia still existed after excluding the impact of prebiotic intake. In addition, the results of the ROC curves we constructed demonstrated the importance of probiotics in the model for predicting hyperuricemia, but unfortunately, the predictive power of the model was shown to be the same, with no significant decrease after removal.

Many experts believe that beneficial gut bacteria provide the host with various nutrients, prevent infections, and regulate immunity, which improves host health (9, 13). Studies have shown that specific gut microbiota can regulate the synthesis and catabolism of purines and uric acid (UA) (6), and conversely, changes in bacterial composition may contribute to the progression of hyperuricemia (20, 21). The prevailing view is that prebiotics and probiotics may alter the gut flora to some extent, thereby reducing the development of hyperuricemia; however, there are fewer extensive cross-sectional studies in this area. In this study, which included 7,176 subjects, logistic regression modeling and calculation of odds ratios confirmed that probiotics play a beneficial role in preventing the development of hyperuricemia and that this effect is not confounded by prebiotic intake.

The preventive effect of probiotics on hyperuricemia may be due to their modulation of the gut flora in favor of the internal environment, which plays a regulatory role in uric acid and purine metabolism (21–24) as a recent study published in CELL has clearly demonstrated the important role of the gut flora in anaerobic uric acid metabolism (25). Several studies have also suggested possible mechanisms in the pathways or influencing metabolism factors. *Lactobacillus*, *Bifidobacterium*, and *Saccharomyces cerevisiae* have been used safely and effectively as probiotics for a long time and are the most studied probiotics (26). Using these probiotics alters the abundance of the gut flora, disrupting the existing balance and shifting the internal environment in a more favorable direction (23, 24, 27). Furthermore, certain probiotics modulate uric acid levels by altering the metabolic balance of amino acids, unsaturated fatty acids, etc. (20, 28, 29) and affect extrarenal excretion by inhibiting the transport process of urate transport proteins (e.g., SLC22A11, SLC17A3, and SLC2A9) in the gut (6, 22, 30). Regulation of uric acid oxidase (uricase), xanthine oxidase (XO), and xanthine dehydrogenase (XDH) activities by different bacterial strains also impair purine absorption and alter the balance of the intestinal flora, which can attenuate or exacerbate hyperuricemia. This has been demonstrated by experimental results with strains of *Lactobacillus fermentum* JL-3 (31), *Lactobacillus rhamnosus* Fmb14 (32), *L. plantarum* (33), and others.

Besides the metabolic factors mentioned above, scientists such as Kim and Hou have suggested that uric acid has a dual role as an antioxidant and an inflammatory promoter, suggesting that immune function is also crucial in the pathogenesis of hyperuricemia and gout (27, 29). A study using a uric acid oxidase knockout (Uox-KO) mouse model with experimentally observed intestinal immune dysregulation and impaired intestinal barrier demonstrated that impaired intestinal integrity contributes to the pathogenesis of HUA. Also, it proposed that the gut microbiota profoundly influences purine nucleotide catabolism and CD4+ Th17 cell infiltration by disrupting amino acid (AA) metabolism, in which CD4+ Th17 cells play an essential role in HUA and gout inflammation (6).

Most studies consider prebiotics to be alternatives to probiotics or additional supplements that stimulate the growth and activity of beneficial bacteria in the gastrointestinal tract, with natural products such as inulin and lactose being essential sources of prebiotics (10). The mechanism of action of prebiotics is not fully understood, with articles suggesting that the health-promoting effects of prebiotics are related to bifidobacteria (26, 34). Meta-analyses have also been conducted to investigate the effects of prebiotics on metabolites such as short-chain fatty acids (SCFAs), but with mixed results (35–37). In our study, the regression models constructed with or without adjustment for covariates yielded OR values less than 1, but the tests were not statistically different, which may indicate that the effect is relatively weak, requires prolonged administration, accumulates in the body at higher concentrations, or is affected by other confounding factors that were not included, such as different intestinal conditions.

Among the subjects enrolled in our study, hyperuricemia tends to have demographic characteristics such as older age, male predominance, a higher proportion of Non-Hispanic White participants, higher BMI, lower PIR, also slightly lower educational level, and more smoking preference, and in terms of disease state, it may be more likely to be comorbid with underlying conditions such as HBP, DM, and CKD. These characteristics are consistent with previous studies of hyperuricemia in the NHANES database (38, 39) and highlight the importance of controlling for covariates when assessing the relationship between probiotic intake and HUA. Several studies further discussed the association between diseases; for example, Krishnan (40) found a non-linear association between hyperuricemia and glomerular function, with prevalence increasing with decreasing glomerular function and that an eGFR of 60 mL/min/1.73 m^2^ appears to be the threshold for a dramatic increase in the prevalence of gout; and several reviews have further examined hypertension, diabetes mellitus, chronic kidney disease and hyperuricemia, and it is generally accepted that hyperuricemia is strongly associated with these diseases and may even be an independent risk factor (41–43). However, a report from a scientific workshop organized by the National Kidney Foundation also points out that these associations need to be supported by additional clinical studies and that routine treatment of hyperuricemia in patients with hypertension, nephropathy, or metabolic syndrome/type 2 diabetes mellitus is not recommended at present (44).

Discussing these factors above, combined with epidemiological studies related to hyperuricemia and gout, can confirm the significant influence of age, gender, race, education level, BMI, etc., on hyperuricemia (45). Meanwhile, there are also some machine learning studies confirming the significant influence of poor lifestyle, abnormally high biochemical indices such as creatinine, lipids, and glucose, as well as hypertension, heart failure, diabetes mellitus, and end-stage renal disease, and other underlying diseases have a notable influence on adverse clinical outcomes in hyperuricemia (46). Therefore, our study also constructed a disease prediction model, calculated AUC values, and plotted ROC curves. The results showed that the value of factors such as age, sex, ethnicity, BMI, HBP, and CKD in predicting hyperuricemia was remarkable. Unfortunately, although the AUC values obtained before and after the addition of probiotics differed and showed better predictive performance, this effect was not statistically significant (AUC: 0.7877 versus 0.7886, *Z* = 1.626, *p* = 0.104). This result suggests that probiotics are not suitable for inclusion in a prediction model for hyperuricemia. Fortunately, there was no significant interference between probiotic consumption and these disease predictors after subgroup analyses.

As with most studies, our study has several limitations. First, we selected data from 2011 to 2018 for cross-sectional analyses to examine associations between prebiotics, probiotics, and hyperuricemia outcomes in the US adult population. This design limited our ability to determine temporality and causality. Second, the data collected were self-reported, which may introduce recall bias and affect the accuracy of the information. Third, the pathogenesis of hyperuricemia is complex, and our study did not adjust for all possible confounders, such as unspecified medication use, dietary habits, or genetic predisposition. These limitations suggest that further longitudinal studies with more controlled variables are needed to understand the relationship between probiotics and hyperuricemia better.

## 5 Conclusion

Our study suggests that probiotic consumption is associated with a reduced risk of hyperuricemia in the US adult population but is not suitable for inclusion in disease prediction models, in contrast to the notable effect of prebiotics on hyperuricemia. However, this finding needs to be confirmed in future longitudinal prospective studies.

## Data Availability

The datasets presented in this study can be found in online repositories. The names of the repository/repositories and accession number(s) can be found in the article/Supplementary material.
