# Supplementary material for: Association between prebiotic, probiotic consumption and hyperuricemia in U.S. adults: a cross-sectional study from NHANES 2011–2018
Source: Front Nutr. 2025 Mar 14;12:1492708. doi: 10.3389/fnut.2025.1492708 (PMC11949776; doi:10.3389/fnut.2025.1492708)
Supplement: Supplementary file 1 [file Table_1.DOCX]

**Table S1. The classification of probiotics and prebiotics**

| Prebiotic | Probiotic |
| --- | --- |
| glucan, gum, arabic, inulin, oligofruc, oligosac, prebiotic, pre-biotic, resistant starch, chicor, psyllium, resveratrol, lactulose | acidophilus, animalis, bacillus, bacilli, bifidobacteri, bifidum, boulardii, breve, brevis, bulgaricus, casei, cerevisiae, coagulans, delbrueckii, enterococcus, faecalis, faecium, fermentum, gasseri, helveticus, infantis, lactis, lactic acid bacteria, lactobacill lactococcus, leuconostoc, licheniformis, longum, mesenteric, paracasei, pediococcus, plantarum, probiotic, pro-biotic, pro biotic, propionibacteri, reuteri, rhamnosus, saccharomyceslivarius, streptococcus, subtilis, thermophilus, E. coli |
